# Pseudopheochromocytoma Associated with Domestic Assault

**DOI:** 10.1155/2016/6580215

**Published:** 2016-09-21

**Authors:** H. M. Le, G. Carbutti, D. Ilisei, E. Bouccin, X. Vandemergel

**Affiliations:** ^1^Department of General Internal Medicine, Centres Hospitaliers Jolimont, Nivelles, Belgium; ^2^Intensive Care Unit, Centres Hospitaliers Jolimont, Nivelles, Belgium

## Abstract

Pseudopheochromocytoma has a clinical presentation that is similar to pheochromocytoma. It manifests itself with paroxysmal hypertension crises, associated with various symptoms such as headaches, chest pain, nausea, palpitations, and dizziness. Patients are usually asymptomatic in between the crises. Unlike pheochromocytoma, there is no catecholamines overproduction in this pathology: hypertensive peaks are caused by a hyperactivation of the sympathetic nervous system, which is often triggered by a psychological trauma in the past. Treatment of pseudopheochromocytoma can be challenging due to normal blood pressure values in between the hypertensive peaks; it includes alpha- and beta-blockers for moderate crises and prevention and must be combined with psychopharmacologic agents such as anxiolytics or antidepressant drugs. Psychotherapy and dietetic treatment are also crucial in pseudopheochromocytoma management.

## 1. Introduction

Severe and symptomatic paroxysmal hypertension always induces suspicion of pheochromocytoma. However, Kuchel described patients with paroxysmal hypertension without catecholamines overproduction [1]. This entity called “pseudopheochromocytoma” is characterized by paroxysmal and abrupt onset of hypertension with symptoms (headache, chest pain, dizziness, nausea, flushing, and palpitations) which is not induced by fear or panic [2]. Moreover in the majority of cases, a past history of severe emotional trauma is found such as physical or psychological violence [2]. We report a case and review the literature about this entity.

## 2. Case Report

A 45-year-old woman was referred to the Emergency Unit by the cardiologist for management and investigation of symptomatic severe hypertension. At admission, blood pressure was high at 190/130 mmHg. Heart rate was normal at 78 bpm. The clinical examination was unremarkable. There was no evidence of aortic dissection and the patient was transferred to the Intensive Care Unit for blood pressure stabilization.

Prior to admission, the patient was seen for the first time by cardiologist because she suffered from symptomatic hypertension with headaches, scotoma, tinnitus, epistaxis, paresthesia in the upper limbs, and dizziness lasting for 4 months. The episodes occurred several times per month but were more frequent of late and tended to happen every 2 days, lasting from a few minutes to an hour. The patient felt well between those episodes except for fatigue. She had been taking a combined antihypertensive drug (angiotensin-conversion enzyme inhibitor with diuretics) for 3 days prescribed by her general practitioner.

Diagnosis of hypertension was established by her GP, confirmed by blood pressure self-measurement, which showed systolic pressure up to 180 mmHg and diastolic pressure up to 80 mmHg. The patient also had palpitations and tachycardia up to 147 per minute.

The patient had no major medical history, although she had depression when her first husband died from a car accident 20 years ago, leaving her in charge of a small child. She has now remarried and had 2 other children. She explained that her son from her first husband had psychological issues and drinking addiction; when drunk, he could be verbally and physically violent towards her. She confessed that her son tried to stab her 4 months ago and went to jail after that event. She had recently started seeing him again but was very anxious during their meetings. She did not drink alcohol but usually smoked a few cigarettes on social occasions. She did not use illicit drugs and did not drink coffee regularly.

Biological results were strictly normal. There was no renal impairment. Hormone determination showed no evidence for secondary hypertension causes such as hypercorticism, hyperthyroidism, hyperaldosteronism, carcinoid tumour, or pheochromocytoma (Table 1). Plasmatic adrenaline and noradrenaline levels were within the normal range but urinary vanillylmandelic acid and urinary normetanephrine levels were slightly high (with, resp., 29.1 mg/24 h (1.8–6.7) and 289 micrograms/g creatinine (46–256)). Urinary catecholamine tests were repeated a few days thereafter and returned to normal.

Cardiac assessment by echocardiography and electrocardiogram was normal; there was no sign of hypertrophy or cavity dilatation, and cardiac output was normal. Cerebral scan revealed no lesion.

An abdominal CT scan was performed and showed no renal artery stenosis or adrenal mass.

Clinical evolution was satisfactory: blood pressure was well controlled by intravenous nicardipine in the first place and then by oral antihypertensive drugs afterwards (ACE inhibitor associated with calcium antagonist). Headaches were treated with simple painkillers. The patient was transferred to the Internal Medicine ward after 5 days spent in the ICU.

Fundoscopic examination of the eye revealed no retinopathy. Magnetic resonance imaging of the brain was normal. ^123^MIBG-scintigraphy was performed and ruled out the existence of adrenal or ectopic secreting mass.

During hospitalization, we observed recurring paroxysmal hypertension crises at 210/120 mmHg despite the treatment. Beta-blockers and diuretics were added to the initial treatment but had little effect. The patient had hypertensive peaks at 240/150 mmHg and subsequently developed a seizure due to a hypertensive encephalopathy; she was readmitted in the ICU for 5 days. It must be noted that there was no orthostatic hypotension.

After excluding every cause of secondary hypertension, pseudopheochromocytoma was the most likely diagnosis for paroxysmal hypertension without evidence of catecholamine excess or organ damage and often appears with an underlying psychological context. Emotional instability can trigger hypertension peaks, which mimic clinical manifestations of pheochromocytoma, even though patients are not aware of their psychological issues.

Adequate treatment was started with alpha- and beta-blockers, combined with anxiolytics and psychological support. Hypertensive crises rapidly resolved after several days and the patient was discharged after normalization of blood pressure.

Two weeks later, the patient was readmitted in the ICU for tonic-clonic seizures with loss of consciousness. At admission, Glasgow Coma Scale was assessed at 3/15 and systolic blood pressure was of 220 mmHg. Electroencephalogram and cerebral scan were normal. Artificial ventilation was needed for a few hours. The patient recovered quickly and was transferred the day after to the General Internal Medicine Unit. The patient recognized afterwards that she had not taken the medication properly at home. Importance of treatment and psychological follow-up was explained again to the patient before discharge. She was seen at medical visit 3 weeks after hospitalization: the patient is now under a psychologist's care, she has no symptoms anymore, and blood pressure is within normal range, which allows progressive decrease of antihypertensive drug doses.

## 3. Discussion

Causes of catecholamines excess include pheochromocytoma, stress, medications, and ingestion of cocaine or amphetamines [3]. The presence of paroxysmal hypertension almost always raises the possibility of a pheochromocytoma and patients with negative evaluation often get labelled with a diagnosis of pseudopheochromocytoma [1].

Clinically, hypertension episodes are usually sudden in onset and are often accompanied by physical symptoms including headaches, dizziness, nausea, diaphoresis, chest pain, and palpitations [2] which are indistinguishable from the symptoms observed in a patient with pheochromocytoma. Frequency of these episodes ranges from daily to less than one per month and the duration of the episodes may range from minutes to days [4]. In their series of 21 patients, Mann found that women are more often affected than men [2] and that the majority of patients had 1–3 attacks per week. The most frequent symptoms were chest pain (80%) followed by light-headedness (68%), headache (68%), nausea, and diaphoresis (62%). Pseudopheochromocytoma is usually described in White Caucasians but case report in Black African is also reported [5].

Some cases had been associated with obstructive sleep apnea [6] or anxiolytic withdrawal [7]. Focal neurological symptoms such as lateralizing sensorimotor deficits were reported in one case [8].

Frequently, a history of psychological trauma is found as in our case. Indeed, four months before admission, the patient's son, under alcohol influence, tried to stab her. The father of her son died in a car accident when he was 25 and her son began to drink some years later. She had depression at that time.

Psychological history includes physical and verbal abuse in childhood [8]. In the series reported by Mann, 67% of patients had a history of severe emotional trauma [2] including physical child abuse or, in another case, the patient said that her son became paraplegic after a nearly fatal automobile crash. As in our case, patients harboured no distressing trauma-related emotions suggestive of emotional defences.

The differential diagnoses are reported as follows:

 Labile hypertension: patients are able to identify a clear emotional or stressful stimulus for blood pressure elevation
 Panic disorder: pressure elevation is usually not as high, characterized by episodes of fear that begin abruptly and not always expected or triggered
 Drug abuse: cocaine, amphetamine, or monoamine oxydase inhibitor
 Isolated adrenal medullary hyperplasia: increased uptake in iodine-131-metaiodobenzylguanidine radioisotope

Labile hypertension differs starkly from paroxysmal hypertension in the clear relationship between blood pressure elevation and stress or emotional distress. Panic disorder is characterized by existence of trigger such as fear or panic, and blood pressure elevation is generally mild. The common point between these entities is the existence of emotions that are repressed from conscious awareness, and both respond to treatment with antidepressant drugs.

Despite the fact that there is no generally accepted definition of stress-related disorders, it was ruled out by psychologist and psychiatrist. The patient did not present dissociative symptoms (no feeling numb or detached, no derealization, no depersonalization, and no dissociative amnesia). Moreover there was no reexperience of the trauma event.

Pathogenesis of pseudopheochromocytoma is poorly understood but is thought to be a result of hyperactivation of the sympathetic nervous system [3]. Patients with pseudopheochromocytoma appear to have an amplified cardiovascular responsiveness to catecholamines and an enhanced adrenal release of epinephrine in response to sympathetic nervous stimulation. Kuchel [1] identified elevated dopamine levels as a marker of sympathetic nervous system activation in pseudopheochromocytoma. In a study of 11 patients with pseudopheochromocytoma, Sharabi et al. [9] found normal baseline plasma concentrations or norepinephrine but higher baseline plasma concentrations of epinephrine and metanephrine. Moreover, these patients had a 6-fold larger increase in plasma epinephrine after stimulation of the sympathetic nervous system with glucagon. Hamada et al. [10] demonstrated increased blood pressure reactivity in patients with pseudopheochromocytoma compared with pheochromocytoma.

In their case report on a patient presenting pseudopheochromocytoma associated with obstructive sleep apnea, Cheezum and Lettieri [6] observed normal value of epinephrine but elevation of total metanephrines and normetanephrine with normalization after continuous positive airway pressure (CPAP) therapy.

Treatment of this condition can be challenging, as blood pressure may be normal in between episodes of high blood pressure. Antihypertensive drugs, psychopharmacologic agents, and psychological interventions are cornerstone of the treatment. Severe elevation of blood pressure may require intravenous drugs such as labetalol or nicardipine. Oral labetalol is less preferred because of the unpredictable bioavailability [11]. Alpha- and beta-blockers are the treatment of choice in case of moderate crisis [2]. Preventive management of blood elevation includes beta-blockers or alpha-blockers. Combination treatment has not been assessed in a controlled trial. Psychopharmacologic agents include rapidly acting anxiolytics, such as Alprazolam [12, 13]. Antidepressant drugs are effective in decreasing the frequency of blood pressure elevation even in patients who do not acknowledge an emotional trigger for these paroxysms [2]. Psychological interventions are also important by reassuring the patients that hypertensive paroxysms are unlikely to cause a sudden cardiovascular event in the absence of extreme blood pressure elevation. In the series published by Mann [2], 3 patients responded to psychological intervention alone without requiring any pharmacological treatment. Despite the absence of randomized trial in pseudopheochromocytoma, nonpharmacologic strategies must be used such as exercise, limiting alcohol consumption, weight loss, and smoking cessation [14]. Meditation merits consideration [15, 16]. Music was also demonstrated effective in some trials [17, 18].

## Figures and Tables

**Table 1:** Laboratory findings: hormonal tests.

Hormonal tests	Values (normal range)
Plasmatic cortisol	<39 nmol/L (126.8–656.5)
Plasmatic aldosterone	14.2 pg/mL (18–232)
Plasmatic renin	12.9 *µ*mU/mL (4–50)
Plasmatic noradrenaline	278.2 pg/mL (100–400 lying, 200–700 standing)
Plasmatic adrenaline	15.4 pg/mL (<50 lying, <100 standing)
Plasmatic dopamine	Nondetectable (0–80)
Urinary normetanephrines	289 *μ*g/g creat (46–256)
Urinary metanephrines	98.8 *µ*g/g creat (27–155)
Urinary vanillylmandelic acid	29.1 mg/24 H (1.8–6.7)
Urinary 5-hydroxyindoleacetic acid	6.8 mg/24 H (0.7–8.2)
